# Microbiological evaluation of LOCATOR® Legacy attachments: A cross‐sectional clinical study

**DOI:** 10.1002/cre2.209

**Published:** 2019-07-18

**Authors:** Ursina Nagy, Christophe Guédat, Catherine Giannopoulou, Martin Schimmel, Frauke Müller, Murali Srinivasan

**Affiliations:** ^1^ Division of Removable Prosthodontics, University Clinics of Dental Medicine University of Geneva Geneva Switzerland; ^2^ Division of Orthodontics, University Clinics of Dental Medicine University of Geneva Geneva Switzerland; ^3^ Division of Periodontology, University Clinics of Dental Medicine University of Geneva Geneva Switzerland; ^4^ Division of Gerodontology, School for Dental Medicine University of Bern Bern Switzerland; ^5^ Service of Geriatrics, Department of Internal Medicine, Rehabilitation and Geriatrics Geneva University Hospitals Geneva Switzerland; ^6^ Clinic of General‐, Special Care‐, and Geriatric Dentistry (KABS), Center of Dental Medicine University of Zurich Zurich Switzerland

**Keywords:** cross‐sectional study, implant overdentures, locator legacy attachments, microbiological evaluation, removable prosthodontics

## Abstract

**Objective:**

This retrospective cross‐sectional study aimed to evaluate quantitatively the oral microbiome in the tri‐lobe central cavity of Locator Legacy attachment and verify whether it harbors a different, potentially more pathogenic, bacterial spectrum than the adjacent edentulous ridge.

**Materials and Methods:**

Edentulous patients rehabilitated with implant overdentures using Locator Legacy attachments were recruited for this study. The clinical examination comprised probing depths, mobility, peri‐implant, and periodontal health along with intraoral swabs for microbiological evaluation, polymerase chain reaction (PCR) testing, and candida culture. The swabs were collected from the trilobed cavity of the attachment and the adjacent edentulous ridge. PCR was performed to detect six specific bacteria, *Porphyromonas gingivalis*, *Aggregatibacter actinomycetemcomitans*, *Tannerella forsythia*, *Treponema denticola*, *Prevotella intermedia*, and *Parvimonas micra*. Statistical analyses were performed using McNemar's test and Wilcoxon's rank sum test with the significance set to *p* < .05.

**Results:**

A total of 50 participants with a mean age of 71.5 ± 9.6 years participated in the study. No significant differences in the microbiome were found between samples from the ridge and the attachment. No significantly different numbers in the candida cultures were identified, and the presence of a removable prostheses did not demonstrate a significant association with the prevalence of candida.

**Conclusions:**

Within the limits of this study and the investigated bacterial species, the trilobed cavity of the attachment does not seem to increase the bacterial load.

## 1 INTRODUCTION

Removable dental prostheses retained by dental implants are associated with high clinical success and patient satisfaction in both partially and completely edentulous subjects (Awad et al., 2003; Awad, Rashid,, & Feine, 2014; Emami, Heydecke, Rompre, de Grandmont, & Feine, 2009). Furthermore, in the age‐advanced elderly patients, implant‐retained removable dental prostheses improve the patient comfort as well as their masticatory efficiency (Awad et al., 2003; Awad, Rashid,, & Feine, 2014; van Kampen, van der Bilt, Cune, Fontijn‐Tekamp, & Bosman, 2004; Visser, Raghoebar, Meijer, Batenburg, & Vissink, 2005). The success of implant overdentures (IODs) and implant‐retained removable partial dentures is multifactorial, but one of the major factors associated with the success depends on the attachment employed (Rutkunas, Mizutani, & Takahashi, 2007; Rutkunas, Mizutani, Takahashi, & Iwasaki, 2011). Stud‐type attachments (unsplinted attachments) are popular for IODs because of their simplicity in clinical and laboratory handling (Davis & Packer, 2000). They are easy to incorporate and do not require elaborate laboratory/clinical procedures for their repair and maintenance (Davis & Packer, 1999; Davis & Packer, 2000; Quirynen et al., 2005). The LOCATOR® Legacy (Zest Dental Solutions) attachments have been documented to be one of the most popular attachments for IODs (Kronstrom & Carlsson, 2017). These attachments have been evidenced with good patient—, as well as clinician— satisfaction while also improving the oral health‐related quality of life of the patients (Cakarer, Can, Yaltirik, & Keskin, 2011; Fernandez‐Estevan, Montero, Selva Otaolaurruchi, & Sola Ruiz, 2017; Kappel, Giannakopoulos, Eberhard, Rammelsberg, & Eiffler, 2016; Mackie, Lyons, Thomson, & Payne, 2011; Zou et al., 2013; Zou, Wu, Huang, Zhang, & Zhang, 2013). However, the LOCATOR® Legacy attachments have also been reported to have a few known disadvantages such as rapid loss of their retention, high maintenance needs, and attachment wear (Al‐Ghafli, Michalakis, Hirayama, & Kang, 2009; Alsabeeha, Atieh, Swain, & Payne, 2010; Evtimovska, Masri, Driscoll, & Romberg, 2009; Kleis, Kammerer, Hartmann, Al‐Nawas, & Wagner, 2010; Kobayashi et al., 2014; Srinivasan, Schimmel, Badoud, et al., 2016; Srinivasan, Schimmel, Kobayashi, et al., 2016).

Most importantly, the LOCATOR® Legacy has been criticized because of its “nuisance factor” (Mackie et al., 2011). The trilobe central cavity present on the attachment head is a frequent site of debris accumulation, when left unremoved, and is a cause of great inconvenience to the elderly patient with compromised vision and manual dexterity. This debris accumulation in the central cavity may impede the insertion of the prosthesis and, especially when unilateral, may cause denture fracture. In addition, such noninsertion may severely impair the oral health‐related quality of life of the patient. Moreover, accumulated debris and biofilm are a potential nidus for propagating oral or more distant general infections. This is of particular importance in the compromised elderly patient who is dependent for care as it could lead to potential complications such as aspiration pneumonia, especially when swallowing disorders are present (Daly et al., 2018; Iinuma et al., 2015; Müller, 2015; Pritchard, Crean, Olsen, & Singhrao, 2017; Yoneyama, Yoshida, Matsui, & Sasaki, 1999).

Therefore, the aim of this cross‐sectional study was to evaluate quantitatively the oral microbiome in the trilobe central cavity of the LOCATOR® Legacy attachment in order to verify if the central cavity harbors a different, potentially more pathogenic, bacterial spectrum than does the adjacent edentulous ridge. A secondary aim was to evaluate an association of the presence of Candida with the presence of removable prostheses. Therefore, the null hypotheses set for this study is that there is no differen

ce in the quantity of the microflora present in the central cavity of the LOCATOR® Legacy attachment and the edentulous ridge and, that the presence of Candida is not related to the presence of a removable prostheses.

## 2 MATERIALS AND METHODS

The study protocol was approved by the appropriate ethical committees in Geneva, Switzerland (CER No. 14‐046). The study is reported according to the Strengthening the Reporting of Observational Studies in Epidemiology (STROBE) guidelines (von Elm et al., 2008).

### 2.1 Study design

The study was designed as a retrospective, single‐center, cross‐sectional clinical study on human subjects.

### 2.2 Study setting

The study was conducted in the removable prosthodontics clinics in a university‐setting dental school. The participants were recruited and examined between May 2014 and November 2014. The participants were recruited according to the following inclusion criteria:

- if they were treated at the university dental clinic and received either a removable partial or complete implant‐retained overdenture using LOCATOR® attachments and the prostheses were present in situ for 12 months or longer;
- if they were restored with microrough surface implants, which were loaded following a conventional loading protocol; and
- if they were living independently.

The participants were excluded if they

- presented with a history of repeated, unjustifiable missed appointments;
- were unable to attend the appointment for health reasons or other causes;
- presented with uncontrolled diabetes;
- presented with a history or with a current oncological condition in the head and neck region; and
- were not willing to participate and/or sign an informed consent.

All patients were clinically examined by two investigators (U. N. and C. G.). For each patient, the peri‐implant health was evaluated by 6‐point probing depth measurements using a standard periodontal probe, mobility (Miller, McEntire, Marlow, & Gellin, 2014), modified bleeding and plaque indices (Mombelli, van Oosten, Schurch, & Land, 1987). These were recorded for the natural teeth (if present) and for the implants with the LOCATOR® attachments.

### 2.3 Endpoint/outcome measures

#### 2.3.1 Polymerase chain reaction

A sterilized paper strip was dipped in the central cavity of the male part of the LOCATOR® abutment and subsequently enclosed in a sterile plastic 0.5‐mL tube. A second sterilized paper strip was wiped on the adjacent edentulous ridge and was deposited in a second plastic 0.5‐mL tube. Both tubes were then taken to the microbiology laboratory for analysis. Genomic DNA was extracted using the GenElute Bacterial Genomic DNA Kit (Sigma‐Aldrich Co., St. Louis, MO, USA) in accordance with the manufacturer's instructions. Polymerase chain reaction (PCR) was performed on each sample to detect six specific bacteria (*Porphyromonas gingivalis* [Pg], A*ggregatibacter actinomycetemcomitans* [Aa], *Tannerella forsythia* [Tf], *Treponema denticola* [Td], *Prevotella intermedia* [Pi], and *Parvimonas micra* [Pm]) using species‐specific primers (Table 1). Dynazyme II DNA Polymerase (FINNZYMES OY, Espoo, Finland) was used as polymerase. PCR was carried out using a GeneAmp® PCR System 9700 (Applied Biosystems, Foster City, CA, USA). For the visualization of the PCR amplificates, 20 μL of PCR products was analyzed by gel electrophoresis on 0.8% standard agarose gels using 1DNA digested with HindIII as molecular mass standards. The bacterial species present were categorized as “absent,” “limited presence,” and “strong presence.”

**Table 1:** Primers for the specific detection of six bacteria

Bacteria	Sequence (5′‐3′)	
*P. gingivalis*	Forward	GAG GGG CAG CAT GAT CTT AG
	Reverse	GTC CGT CTT TCA ACG GGT TA
*A. actinomycetemcomitans*	Forward	GGG GAT GTA CTG ACG CTG AT
	Reverse	ACC AGG GCT AAA CCC CAA TC
*T. forsythia*	Forward	GGG TGA GTA ACG CGT ATG TAA CCT
	Reverse	GCC CAT CCG CAA CCA ATA AA
*T. denticola*	Forward	CGT TCC TGG GCC TTG TAC A
	Reverse	TTC ACC CTC CTT ACC AAA CG
P. intermedia	Forward	CAA GTA GCG TGC AGG ATT GA
	Reverse	CCG GTC CTT ATT CGA AGG GTA
P. micra	Forward	TCG AAC GTG ATT TTT GTG GAA A
	Reverse	GGT AGG TTG CTC ACG TGT TAC TCA

#### 2.3.2 Candidiasis test

A cotton swab designed to collect samples from the oral mucosa was rubbed on the tongue and on the insides of the cheek of the participant. The smear was applied to a chromogenic medium for the selective isolation of yeasts and the direct identification of Candida albicans. The different types of colonies were identified after incubation (48 hr at room temperature, without direct light), and the presence of C. albicans was quantified by means of an agar‐type chromID™ Candida (CAN2) by bioMérieux (France).

### 2.4 Study protocol

Patients were recruited from the university clinics of dental medicine. An electronic search of the dental school's patient management software using the key word “LOCATOR®” was used to formulate an initial screening list of prospective patients. The selected patients were then sent a letter of invitation requesting them to participate in a clinical study. Following the letter, they were then contacted by telephone 10–14 days later to answer potential questions on the informed consent and, if they agreed, subsequently fix an appointment for consultation. After an initial screening, the willing participants signed the informed consent. As a first step, the patient's history and the relevant personal information were collected by the two investigators (U. N. and C. G.). The participants were then clinically examined, and all the information was duly recorded in the clinical record form. The examination began with the candidiasis test, before removing the patients' partial or total prostheses. After removing the prostheses, swabs of the edentulous ridge and the LOCATOR® were made for the PCR analyses. The assessment of the periodontal and peri‐implant health parameters were then recorded. No treatment was performed in this study. If a treatment need was identified during the clinical examination, the study participants were informed and were then subsequently referred to their dentist or to a specialist clinician for the appropriate care.

### 2.5 Statistical analysis

Potential quantitative differences between the microbiome of both locations (i.e., the edentulous ridge and the LOCATOR® Legacy attachment) were first verified for each bacterium using contingency tables. The association of the amount of bacteria present on the two different locations was compared and was assessed using McNemar's test. In a second step, for each location, the pattern of bacteria present (e.g., Aa absent, Tf present, Pi absent, Pg absent, Td absent, and Pm absent) was determined. As six different bacteria were assessed by considering their presence as a yes or a no, there were potentially 64 patterns on the ridge and 64 on the attachment. The patterns were compared on both locations using McNemar's test. As a third step, the number of patterns were reduced to the two most frequent patterns, along with a third pattern corresponding to the other remaining potential patterns. The patient and clinical characteristics were then compared among these patterns for both the ridge and LOCATOR® locations using Wilcoxon's rank sum test.

## 3 RESULTS

**Figure 1:**
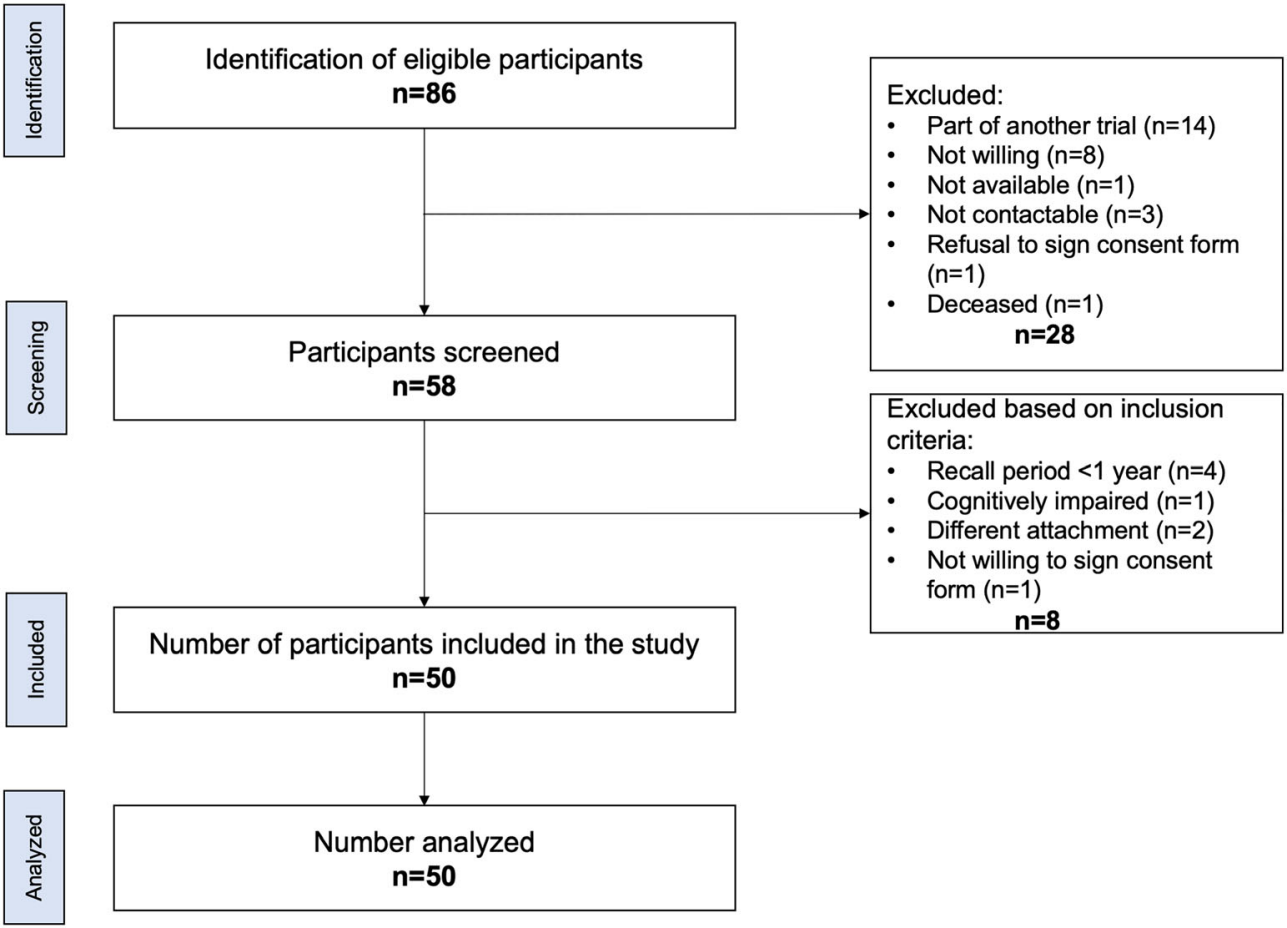
Flow diagram showing the details of the participant identification, screening, and recruitment process

A total of 50 patients (27 women, 23 men) with a mean age of 71.5 ± 9.6 years met the inclusion criteria and participated in the study. The detailed participant demographics has been described elsewhere (Guedat, Nagy, Schimmel, Muller, & Srinivasan, 2018). The details of the participant screening and recruitment process are shown in Figure 1. The incidence (number and frequency) of the studied bacteria on the ridge and on the attachment are listed in Table 2. No different frequencies were found between samples from the ridge and the LOCATOR®. All samples tested negative for Aa; therefore, this bacterium was not considered for the further analyses.

**Table 2:** Incidence number and (frequency) of the studied bacteria in the ridge and LOCATOR®

Bacteria	Ridge	LOCATOR®
Aa	0	0
Tf	35 (70)	33 (66)
Pi	11 (22)	16 (32)
Pg	48 (96)	47 (94)
Td	8 (16)	10 (20)
Pm	3 (6)	5 (10)

Abbreviations: Aa, *Aggregatibacteractinomycetemcomitans*; Tf, *Tannerella forsythia*; Pi, *Prevotella intermedia*; Pg, *Porphyromonas gingivalis*; Td, *Treponema denticola*; Pm, *Parvimonas micra*.

The amounts of Tf, Pi, Td, and Pm were similar in the samples from the ridge and from the LOCATOR® (Table 3). For instance, for Tf, out of 50 patients, 24 (nine with no bacteria, five with some Tf, and 10 with a large amount of Tf) showed an agreement between the ridge and the LOCATOR®. However, the Pg amount differed between the ridge and the LOCATOR® (*p* = .03). Although 31 patients had similar amounts in both locations, 14 patients had large amounts of Pg on the LOCATOR® but low or absent quantities on the ridge, whereas only five patients have more Pg on the ridge than on the LOCATOR®.

**Table 3:** Contingency tables between bacteria in the ridge and LOCATOR® locations

Bacteria	Bacterial load (0 = absent, 1 = limited presence, 2 = strong presence)				p‐value
	Ridge	LOCATOR®			
		0	1	2	
Tf	0	9	5	1	0.60
	1	6	5	8	
	2	2	4	10	
Pi	0	33	7	0	1.00
	1	1	5	2	
	2	0	0	2	
Pg	0	1	0	1	0.03
	1	2	9	13	
	2	0	3	21	
Td	0	39	3	0	1.00
	1	1	2	3	
	2	0	2	0	
Pm	0	45	2	0	0.50
	1	0	3	0	
	2	0	0	0	

Abbreviations: Tf, *Tannerella forsythia*; Pi, *Prevotella intermedia*; Pg, *Porphyromonas gingivalis*; Td, *Treponema denticola*; Pm, *Parvimonas micra*.

Because all patients tested negative for Aa, 32 patterns of the five remaining bacteria were possible. However, only 14 patterns were present on the LOCATOR®, versus 11 on the ridge. Two patterns were particularly frequent in both locations. One was having only Pg, and the other was having both Tf and Pg present. These patterns were similar in both locations (Table 4; *p* = .20).

**Table 4:** Association of patterns in LOCATOR® and ridge

LOCATOR®	Ridge		
	Pg only	Tf and Pg	Other
Pg only	6	5	1
Tf and Pg	1	13	2
other	3	5	14

Abbreviations: Pg, *Porphyromonas gingivalis*; Tf, *Tannerella forsythia*.

Patient characteristics, such as age and sex, were similar across these three patterns, on the ridge and on the LOCATOR®. The presence of a removable prostheses was not associated with a higher prevalence of Candida.

## 4 DISCUSSION

The primary aim of the study was to compare detection frequencies and levels of six pathogenic microorganisms in the trilobe central cavity of the LOCATOR® Legacy attachment and the adjacent edentulous ridge. With the exception of one bacterium *(*Aa was absent in all samples), our results showed that both the ridge and the LOCATOR® were similarly colonized by the studied periodontal pathogens. The most commonly detected bacteria in both locations were Tf and Pg. To the best of our knowledge, this is the first study to compare the microflora on LOCATOR® attachments of elderly subjects wearing removable partial or complete implant‐retained overdentures.

Several studies have shown that after complete loss of teeth, some of the above‐mentioned target species still remain in the oral cavity (Cortelli et al., 2008; Cortelli et al., 2008; Fernandes et al., 2010). Therefore, not only teeth but also the oral soft tissues could act as important reservoirs of bacteria. Andjelkovic et al. (2017) aimed to compare the composition of oral microflora before and after rehabilitation by studying the changes in the prevalence of six common periodontal pathogens in elderly edentulous patients wearing complete dentures (Andjelkovic et al., 2017). Not only were the pathogens present before inserting the dentures, but their prevalence increased considerably during the 6 months that the dentures were worn. At the same time point, co‐associations between bacteria were observed. It is important to emphasize that these bacteria were present in high amounts despite adequate oral hygiene and proper storage of the dentures.

C. albicans by its capability to adhere to mucosal surfaces has been shown to contribute to the pathogenesis of oral candidiasis (McIntyre, 2001). In our study, the high prevalence of C. albicans was not associated with the presence of a removable prosthesis. The study of Kilic et al. (2014) aimed to elucidate the difference between LOCATOR®‐ and bar‐retained overdentures in the prevalence of denture‐related stomatitis and the colonization by Candida species (Kilic et al., 2014). The authors reported higher colony forming unit values of Candida species in the bar‐retained overdentures as compared to those retained by LOCATOR®. Furthermore, the presence of gingival inflammation and plaque increased the prevalence of denture‐related stomatitis, emphasizing the importance for regular denture‐ and attachment‐ surface hygiene. In the same study, the authors observed that C. albicans was the most common species in both bar‐retained and LOCATOR®‐retained overdentures (81.3% vs. 38.1%), followed by Candida glabrata (37.5% vs. 23.8%, respectively).

With the increasing numbers of the old and very old patients receiving implant treatment, hygienic aspects of implant design become more important. Physiological aging includes impaired vision and tactile sensitivity, indicating a lower ability to notice biofilm on natural teeth, dental prostheses, and implant attachments (Boss & Seegmiller, 1981; Janssens, Pache, & Nicod, 1999; Weinstein & Anderson, 2010). Age‐related impairment of manual dexterity precludes further, a meticulous removal of the biofilm that forms with time on any hard object in the oral cavity. Consequently, elderly patients often present with poor oral hygiene and a substantial bacterial load in the oral cavity (Andersson, Renvert, Sjogren, & Zimmerman, 2017; Pritchard et al., 2017). In younger persons, the morphology of a natural dentition is “self‐cleaning,” as the interproximal spaces are filled with gingival papillae and the gingival margin is located near the cemento‐enamel junction. Young persons also rub the oral cavity clean during a meal by using the tongue and the cheeks. This muscle activity helps also in repositioning the food bolus on the oral cavity and/or pushing the food stuffs onto the tongue for a better taste sensation. With age, the forceful chewing and rubbing of the tongue and cheeks diminish substantially, as muscles atrophy and weaken with age and motor coordination becomes more erratic (Campbell, McComas, & Petito, 1973; Newton, Abel, Robertson, & Yemm, 1987; Newton, McManus, & Menhenick, 2004; Newton & Yemm, 1986; Newton, Yemm, Abel, & Menhinick, 1993; Roberts et al., 2016). These age‐related changes explain the abundant presence of biofilm in the elderly persons' mouths and dentitions. Age‐adequate dental restorations need to consider these age‐related functional impairments and require a design, which facilitates the “self‐cleaning.” The central cavity in the LOCATOR® attachment is a functional necessity, as it allows insertion, tightening, and removal of the attachment with the corresponding instrument. The nylon insert of the LOCATOR® does not fully engage into this central cavity, leaving some space, notably the circular undercut “empty.” This volume presents a warm (37°), humid, and dark environment, which intuitively seems a favorable environment for bacterial growth. Clinical experience confirms that in nearly all LOCATOR® attachments a white biofilm is present, as the shape of the small cavity, with its circular undercut, is difficult to clean for the denture wearer. Hence, the aim of this study was to verify if the central cavity presents a particular risk for the accumulation of a—potentially more pathogenic—biofilm deposit, when compared to the adjacent edentulous ridge, where biofilm would be more easily cleared away by the action of the tongue and the saliva, where it could be more easily removed by a regular tooth brush. Bacterial load from the oral cavity, be it on the natural dentition or the dental prostheses, or dental implants, or even tongue coating, presents a risk of developing aspiration pneumonia (Abe, Ishihara, Adachi, & Okuda, 2008; Awano et al., 2008; Kageyama et al., 2018). The evinced risk factors include pocket depths of more than 5 mm, poor oral hygiene, nocturnal denture wearing, and the presence of swallowing disorders. The swallowing reflex requires a complex coordination of various motor patterns to assure a smooth transition from one phase of deglutition to the next. With motor coordination being affected by the aging process, the swallowing reflex more often “trips over” in the elderly, leading to the aspiration of saliva and food stuffs (Schmidt, Holas, Halvorson, & Reding, 1994). The prevalence of swallowing disorders increases from 6–9% in the adult population to 15–22% in persons aged 50 years or older and reaches 40–60% in institutionalized elders (Aslam & Vaezi, 2013).

Pneumonia is one of the major threats for the aged population with an estimated incidence of 33 to 114 cases for 1000 population per year for persons living in institutions (Janssens & Krause, 2004). Pneumonia is the leading cause of all infections in nursing homes and the leading cause of death from infection in patients aged 65 years and older (El‐Solh, 2011a; El‐Solh, 2011b). Bacteria from the oral cavity, corresponding to the periodontal microbial flora, was identified from the bronchoalveolar sputum retrieved from the broncholavage in hospitalized elderly pneumonia patients (Imsand, Janssens, Auckenthaler, Mojon, & Budtz‐Jorgensen, 2002; Quagliarello et al., 2005), confirming the contribution of the oral microbiome. Further evidence for a causal contribution of the periodontal bacteria arises from randomized controlled trials indicating a reduced incidence of pneumonia if weekly oral hygiene is practiced by dental personnel, such as hygienists or dentists (Andersson et al., 2017; Sjogren, Nilsson, Forsell, Johansson, & Hoogstraate, 2008; Sjogren, Wardh, Zimmerman, Almstahl, & Wikstrom, 2016). Even taking a removable prosthesis out during the night might reduce the microbiological burden and showed consequently a reduced risk for developing pneumonia, when compared with habitual nocturnal denture wearing (Iinuma et al., 2015).

Given the above‐mentioned evidence on a frequently poor oral hygiene and its potential impact on an elderly person's well‐being, it seems particularly important to verify if dental restorations, which are integrated into the oral cavity and coated with oral biofilm shortly after insertion, will not introduce a novel risk for bacterial load. The results from this present study confirm that the central cavity on the LOCATOR® attachment does not lead to a different bacterial spectrum quantitatively. Hence, using the LOCATOR® attachment does not present a risk for a changed/increased oral bacterial flora. However, the issue with the mechanical obstruction of the trilobe cavity with oral debris still remains. This might impede prosthesis insertion. The problem can be prevented by filling the central cavity with a provisional composite restoration that can be easily removed on demand. However, these fillings might also harbor microorganisms as they are not definitively bonded and hence do not provide a perfect marginal seal to prevent percolation of oral fluids and bacteria. Therefore, a design change would be a valid approach to reduce or eliminate this trilobed cavity to prevent complications.

Although this study has been conducted with sound methodology adhering to strict guidelines, certain weaknesses do exist. The sample size in this study was small and could have influenced the results. Perhaps a larger sample size could have elicited a significant difference. However, the limitation related to the sample size calculation could not have been prevented, as studies evaluating similar outcomes are not available in current literature. Moreover, the study cohort, with regard to the type of prosthesis, number of implants supporting the prosthesis, and the jaw of rehabilitation, was heterogenous. An adequate number of the recruited participants with similar characteristics could not be segregated into relevant participant groups, for a more detailed analysis. All the participants were pooled into one group and then analyzed. This could also have undermined the results. Nevertheless, the sample size of 50 with similar implants and attachments retaining some form of removable dental prostheses was considered an acceptable number for the analysis of the endpoints outlined in this study. Another shortcoming of the study concerns the number of the bacteria studied that were limited to six. By using the conventional PCR method, only the expected bacteria were detected by using specific primers. More sophisticated methods, such as broad‐range PCR or pyrosequencing, may have allowed a much higher number of bacteria to be studied and perhaps may have revealed significant differences between the ridge and the LOCATOR®. Furthermore, factors related to the participant that could have contributed to the colonization of bacteria, such as systemic health conditions and medications, manual dexterity, cognitive status, functional independence measures, depression, frailty, dependence for instrumental activities of daily living, and dietary habits, were not assessed and analyzed. Perhaps including these confounders could have added more to the interpretation of the results. However, the findings of the study do help conclude that the LOCATOR® Legacy attachment does not effectively augment the studied bacterial species.

## 5 CONCLUSION

The results of the study confirm that the trilobed cavity present on the LOCATOR® Legacy attachment head does not seem to be introducing a novel bacterial spectrum or an increased bacterial load. However, this conclusion cannot be extrapolated beyond the investigated six bacterial species.

## CONFLICT OF INTEREST

None.
